# The effectiveness of antiepileptic drug treatment in glioma patients: lamotrigine versus lacosamide

**DOI:** 10.1007/s11060-021-03800-z

**Published:** 2021-07-01

**Authors:** Mark P. van Opijnen, Pim B. van der Meer, Linda Dirven, Marta Fiocco, Mathilde C. M. Kouwenhoven, Martin J. van den Bent, Martin J. B. Taphoorn, Johan A. F. Koekkoek

**Affiliations:** 1 Department of Neurology, Leiden University Medical Center, PO BOX 9600, 2300 RC Leiden, The Netherlands; 2 Department of Neurology, Haaglanden Medical Center, The Hague, The Netherlands; 3 Department of Biomedical Data Sciences, Medical Statistics, Leiden University Medical Center, Leiden, The Netherlands; 4 Mathematical Institute, Leiden University, Leiden, The Netherlands; 5 Department of Neurology, Amsterdam University Medical Centers, location VUmc, Amsterdam, The Netherlands; 6 Department of Neurology, Erasmus Medical Center, Rotterdam, The Netherlands

**Keywords:** Glioma, Epilepsy, Lamotrigine, Lacosamide, Antiepileptic drug, Treatment failure

## Abstract

**Purpose:**

Optimal treatment with antiepileptic drugs (AEDs) is an important part of care for brain tumor patients with epileptic seizures. Lamotrigine and lacosamide are both examples of frequently used non-enzyme inducing AEDs with limited to no drug-drug interactions, reducing the risk of unfavorable side effects. This study aimed to compare the effectiveness of lamotrigine versus lacosamide.

**Methods:**

In this multicenter study we retrospectively analyzed data of patients with diffuse grade 2–4 glioma with epileptic seizures. All patients received either lamotrigine or lacosamide during the course of their disease after treatment failure of first-line monotherapy with levetiracetam or valproic acid. Primary outcome was the cumulative incidence of treatment failure, from initiation of lamotrigine or lacosamide, with death as competing event, for which a competing risk model was used. Secondary outcomes were uncontrolled seizures after AED initiation and level of toxicity.

**Results:**

We included a total of 139 patients of whom 61 (44%) used lamotrigine and 78 (56%) used lacosamide. At 12 months, there was no statistically significant difference in the cumulative incidence of treatment failure for any reason between lamotrigine and lacosamide: 38% (95%CI 26–51%) versus 30% (95%CI 20–41%), respectively. The adjusted hazard ratio for treatment failure of lacosamide compared to lamotrigine was 0.84 (95%CI 0.46–1.56). The cumulative incidences of treatment failure due to uncontrolled seizures (18% versus 11%) and due to adverse events (17% versus 19%) did not differ significantly between lamotrigine and lacosamide.

**Conclusion:**

Lamotrigine and lacosamide show similar effectiveness in diffuse glioma patients with epilepsy.

**Supplementary Information:**

The online version contains supplementary material available at 10.1007/s11060-021-03800-z.

## Introduction

Gliomas are the most common malignant primary brain tumor. The median overall survival depends on several factors, such as World Health Organization (WHO) tumor grade, preoperative Karnofsky Performance Status (KPS), age, and extent of surgical resection [1]. Despite multimodal treatment strategies, the prognosis still remains poor with a high recurrence rate [2–4]. Patients may suffer from generic cancer symptoms (e.g. fatigue and pain), but also from central nervous system specific symptoms (e.g. mood disorders, focal neurological or cognitive deficits, and seizures). Epileptic seizures are frequently reported in glioma patients with incidences up to 90%, depending on tumor grade, molecular-genetic subtype and location [5–7]. Antiepileptic drugs (AEDs) are the mainstay in the management of seizures, in addition to antitumor treatment with surgery, radiotherapy, and chemotherapy [8].

Optimal AED therapy for patients with brain tumor-related epilepsy (BTRE) is not straightforward, as it may be complicated by pharmacoresistance, adverse effects and drug-drug interactions [7, 9]. Although evidence-based recommendations based on high-quality effectiveness studies are lacking in patients with glioma, there is a general consensus to avoid enzyme-inducing AEDs. Currently, levetiracetam and valproic acid are two of the most frequently prescribed drugs as first-line treatment of epilepsy in glioma patients [10–12]. Within 12 months of initiation, 33% and 50% of patients on first-line monotherapy with levetiracetam or valproic acid, respectively, failed on these drugs due to uncontrolled seizures, adverse events or for other reasons, and a second drug needed to be initiated as alternative or add-on therapy [13]. Although there is no consensus with regard to the preferred AED if a previous AED has failed, lamotrigine and lacosamide are regularly considered. Both are non-enzyme inducing AEDs with limited to no interactions with systemic agents and fewer adverse effects compared to first-generation AEDs, such as carbamazepine, phenobarbital, and phenytoin [9]. Lamotrigine has proven to be effective in non-BTRE, both as monotherapy and as add-on therapy [14–18]. Lacosamide can significantly improve seizure control and is well tolerated as add-on therapy in the non-BTRE population too, therefore being frequently prescribed to patients with glioma [19–21]. Currently, there are no studies that have compared the effectiveness of lamotrigine versus lacosamide in glioma patients with epilepsy.

In studies on drug effectiveness, treatment failure rates (or its inverse: retention rates) are an important outcome, generating a reliable measure for both AED efficacy and tolerability [22]. An AED treatment is failing if a patient discontinues the drug, or if another AED is added to the current AED. The calculation of AED treatment failure rates in glioma patients is complicated because of their generally poor prognosis. Many patients die before reaching the outcome of interest (i.e. AED treatment failure), making death a competing risk in the analysis to calculate treatment failure rates, that needs to be accounted for [23]. This retrospective observational cohort study aimed to compare the effectiveness of AED treatment with lamotrigine versus lacosamide in patients with epilepsy due to a diffuse glioma, by evaluating the treatment failure rates of uncontrolled seizures and adverse events.

## Methods

### Study population and procedures

The study population consisted of adult patients who were diagnosed with a histologically confirmed supratentorial WHO grade 2–4 glioma according to the WHO 2016 guidelines and had undergone surgical biopsy or (re)resection in Haaglanden Medical Center, Erasmus MC Cancer Institute or Amsterdam University Medical Centers between Jan 1st, 2004 and Jan 1st, 2018. All patients were diagnosed with BTRE and received first-line monotherapy treatment with levetiracetam or valproic acid. For the current study, patients who were prescribed lamotrigine or lacosamide during the course of their disease, whether in combination with one or multiple AEDs, or as monotherapy, were eligible. The institutional review boards of all institutions approved the study. Glioma patients without epilepsy who were prescribed prophylactic anticonvulsant treatment and patients with an unknown start date of the AED treatment were excluded from analysis.

Sociodemographic and clinical data of included patients were extracted retrospectively. In this study, baseline refers to the starting date of AED therapy with lamotrigine or lacosamide. We collected age and sex, KPS, date of radiological diagnosis, molecular and histological parameters, tumor grade, radiologic progressive disease before baseline and during follow-up, tumor location, extent of resection, and information on antitumor treatment (i.e. starting date of radio- and/or chemotherapy and type of chemotherapeutic agent). Also, information on seizure type (focal or focal to bilateral tonic–clonic), as well as the start and end date(s) of prescribed AED(s), dosages and, if applicable, reason for AED treatment failure were registered. In case of treatment failure due to adverse events (AEs), type and grade of the AEs were extracted, as well as whether the AEs improved after changing the AED treatment. The toxicity, i.e. grades of AEs, was based on the Common Terminology Criteria for Adverse Events (CTCAE) [24]. In case a patient switched from lamotrigine to lacosamide or vice versa, only data on the first initiated AED until treatment failure was collected. Since lamotrigine and lacosamide have an equal defined daily dose (DDD), i.e. both 0.3 g according to the WHO-index [25], we did no calculate a separate AED load to compare dosages between both AEDs. Dosages were compared to evaluate optimal seizure control in both groups. The protocol was approved by the medical ethics committee of each institution and consent of patients was obtained according to the institution’s policy.

### Outcomes

The primary outcome in this study was treatment failure rate, which reflects the effectiveness of AED treatment by encompassing both AED efficacy and tolerability [18, 23]. The main reasons for treatment failure are uncontrolled seizures or intolerable AEs. Treatment failure due to uncontrolled seizures is defined as any change (i.e. discontinuation of lamotrigine or lacosamide, or addition of another AED) in AED management. Intolerable AEs are defined as treatment related events that resulted in discontinuation of lamotrigine or lacosamide, therefore regarding those cases as treatment failures. Secondary outcomes were: (1) uncontrolled seizures after initiation of lamotrigine or lacosamide, reflecting efficacy, and (2) grade of toxicity, reflecting tolerability. Whether AEs improved or not after discontinuation of lamotrigine or lacosamide was used in order to validate the causality between the treatment failure and AEs. The maximum follow-up was 36 months. Post-drop-out information (i.e. date of death) was used if available in case patients were lost due to progressive disease. If patients were lost to follow-up ≤ 3 months before death, they were considered to have continued the current AED until the date the patient deceased. The following reasons were not considered treatment failure: any dose adjustments of the evaluated AED, addition of an AED taken only if necessary, addition of an AED with a different indication than epileptic seizures, temporary perioperative AED prophylaxis, replacement with a non-oral AED in the end-of-life phase due to swallowing difficulties, or poor adherence less than one week.

### Statistics

Sociodemographic and clinical characteristics between patients in the initial cohort [13] and those included in this study (i.e. using lamotrigine or lacosamide) were compared by using chi-square test for categorical variables and t-test for continuous variables. In case of violation of the normality assumption a non-parametric test was used for the continuous variables. Kaplan–Meier curves were used to calculate time to events of interest. To estimate the cumulative incidence of treatment failure due to uncontrolled seizures or AEs, a competing risk model with two competing risks, treatment failure and death, was used [26]. To assess the difference between cumulative incidences the Gray’s test was used [27]. For secondary outcomes, treatment failure was further divided into four competing risks: treatment failure due to uncontrolled seizures, due to AEs, due to other reasons (encompassing withdrawal due to remission of seizures and unknown reasons), or death. Then, the cumulative incidence for each event was estimated. To estimate the effect of prognostic factors on the two competing events treatment failure and death, cause specific Cox proportional hazard models were estimated. The proportional hazards assumption was checked by looking at the Schoenfeld residuals, nonlinearity by Martingale residuals, and influential observations by deviance residuals. The following potential confounding variables were considered: age (≤ 40 year versus > 40 year), sex, KPS (≥ 70 versus < 70), tumor grade [low grade (WHO grade 2) versus high grade (WHO grade 3–4)], IDH-mutation status, surgical resection (i.e. partial or gross total resection versus biopsy only), prior radiotherapy, prior chemotherapy, tumor involvement in temporal or in frontal lobe, history of psychiatric disorder (i.e. depression, anxiety, or psychotic disorder), and seizure type (i.e. focal or focal to bilateral tonic–clonic). Statistical analyses were performed using statistical package *SPSS* version 26.0. All analysis concerning competing risk were performed in *R*, an open software environment. To estimate the cumulative incidence, the library *cmprsk* was used [26]. P-values < 0.05 were considered statistically significant.

## Results

### Patient characteristics

Population characteristics are described in Table 1. In total, 139 patients were eligible of whom 61 (44%) were prescribed lamotrigine and 78 (56%) lacosamide. Patients on lacosamide compared to lamotrigine were more often male [69% (54/78) versus 44% (27/61), respectively, p = 0.003], and received more often radiotherapy [74% (58/78) versus 49% (30/61), p = 0.002] and systemic therapy [65% (51/78) versus 38% (23/61), p = 0.001]. Also, more patients in the lacosamide group had tumor involvement in the temporal lobe [63% (49/78) versus 46% (28/61), p = 0.046]. After a maximum of 36 months follow-up, in total, 35% (49/139) patients had died: 25% (15/61) in the lamotrigine group, compared to 44% (34/78) in the lacosamide group (p = 0.067). The median time from date of diagnosis to initiation of lamotrigine or lacosamide was 16 months (IQR = 35) and 21 months (IQR = 47), respectively (p = 0.268). Of the patients on lamotrigine, 41% (25/61) had developed progressive disease before starting lamotrigine, while this was 58% (45/78) in patients on lacosamide (p = 0.051). Within the three months before the start of lamotrigine or lacosamide, this difference was 16% (10/61) versus 35% (27/78), respectively (p = 0.016).

**Table 1:** Study characteristics

	Lamotrigine *n* = 61	Lacosamide *n* = 78	Total cohort *n* = 139	p-value
Gender, male, no. (%)	27 (44%)	54 (69%)	81 (58%)	0.003
Age group, no. (%)				0.139
≤ 40 year	21 (34%)	18 (23%)	39 (28%)	
> 40 year	40 (66%)	60 (77%)	100 (72%)	
KPS, no. (%)				0.056
≥ 70	61 (100%)	71 (91%)	132 (95%)	
< 70	0 (0%)	5 (6%)	5 (5%)	
Unknown	0 (0%)	2 (3%)	2 (1%)	
WHO diagnosis, no. (%)				0.138
Grade 2	31 (51%)	31 (40%)	62 (45%)	
Diffuse astrocytoma NOS	12 (20%)	5 (6%)	17 (12%)	
Diffuse astrocytoma IDH-mutant	5 (8%)	12 (15%)	17 (12%)	
Oligodendroglioma NOS	7 (12%)	5 (6%)	12 (9%)	
Oligodendroglioma IDH-mutant 1p/e19q codeletion	7 (12%)	7 (9%)	14 (10%)	
Oligoastrocytoma NOS	0 (0%)	2 (3%)	2 (1%)	
Grade 3	13 (21%)	11 (14%)	24 (17%)	
Anaplastic astrocytoma NOS	4 (7%)	3 (4%)	7 (5%)	
Anaplastic astrocytoma IDH-mutant	3 (5%)	2 (3%)	5 (4%)	
Anaplastic oligodendroglioma NOS	4 (7%)	3 (4%)	7 (5%)	
Anaplastic oligodendroglioma IDH-mutant 1p/19q codeletion	2 (3%)	2 (3%)	4 (3%)	
Anaplastic oligoastrocytoma NOS	0 (0%)	1 (1%)	1 (1%)	
Grade 4	17 (28%)	36 (46%)	53 (38%)	
Diffuse astrocytoma wildtype	2 (3%)	2 (3%)	4 (3%)	
Anaplastic astrocytoma wildtype	0 (0%)	4 (5%)	4 (3%)	
Glioblastoma NOS	10 (16%)	12 (15%)	22 (16%)	
Glioblastoma wildtype	5 (8%)	16 (21%)	21 (15%)	
Glioblastoma IDH-mutant	0 (0%)	2 (3%)	2 (1%)	
Surgical resection, yes, no. (%)	49 (80%)	57 (73%)	106 (76%)	0.461
Radiotherapy, yes, no. (%)	30 (49%)	58 (74%)	88 (63%)	0.002
Systemic therapy, yes, no. (%)	23 (38%)	51 (65%)	74 (53%)	0.001
Systemic therapy detailed, no. (%)				
TMZ (+ additional agents)	18 (78%)	38 (75%)	56 (76%)	
PCV (+ additional agents)	3 (13%)	6 (12%)	9 (12%)	
TMZ + PCV	1 (4%)	7 (14%)	8 (11%)	
Other	1 (4%)	0 (0%)	1 (1%)	
PD before lamotrigine/lacosamide initiation, yes, no. (%)	25 (41%)	45 (58%)	70 (50%)	0.051
PD during lamotrigine/lacosamide, yes, no. (%)	28 (46%)	37 (47%)	65 (47%)	0.857
Number of treatment failures before lamotrigine/lacosamide initiation, median (IQR)	2 (1)	2 (2)	2 (2)	0.243
Median time to lamotrigine/lacosamide, months (IQR)^a^	16.1 (35.3)	20.6 (47.4)	17.7 (39.6)	0.315
Median time to radiotherapy, months (IQR)^3^	15.3 (41.5)	12.5 (22.5)	14.0 (29.5)	0.253
Median time to systemic therapy, months (IQR)^3^	12.1 (26.4)	13.1 (20.0)	12.4 (19.8)	0.785
First-line AED monotherapy started, no. (%)				0.008
Levetiracetam	27 (44%)	52 (67%)	79 (57%)	
Valproic acid	34 (56%)	26 (33%)	60 (43%)	
Lamotrigine/lacosamide combined with another AED, yes, no. (%)	40 (66%)	55 (71%)	95 (68%)	0.534
Tumor in temporal lobe, yes, no. (%)	28 (46%)	49 (63%)	77 (55%)	0.046
Tumor in frontal lobe, yes, no. (%)	40 (66%)	59 (76%)	99 (71%)	0.193
Seizure type, no				0.662
Focal	24 (39%)	27 (35%)	51 (37%)	
Focal to bilateral tonic-clonic^b^	36 (59%)	48 (62%)	84 (60%)	
Unknown	1 (2%)	3 (4%)	4 (3%)	

*IQR* interquartile range; *No.* number of patients; *PD* progressive disease; *SD* standard deviation; *KPS* Karnofsky Performance Status; *WHO* World Health Organization; *TMZ* temozolomide; *PCV* Procarbazine, lomustine and vincristine

^a^Calculated from date of radiological diagnosis

^b^Patients had either solely focal to bilateral tonic–clonic seizures or both focal and focal to bilateral tonic–clonic seizures

Baseline sociodemographic and clinical characteristics of patients included in this study were compared to the baseline of patients who were not prescribed lamotrigine or lacosamide (*n* = 1296). Patients in the current study were significantly younger, had a higher KPS, and more often had a lower WHO grade (see Supplemental S1), reflecting that this is a selected population with more difficult to treat seizures.

### AED treatment

Patients used either levetiracetam or valproic acid as a first-line AED, and significantly more patients in the lamotrigine group were initially prescribed valproic acid (56%, 34/61) compared to patients in the lacosamide group (33%, 26/78), p = 0.008. Number of failures on other AEDs before switching to either lamotrigine or lacosamide was comparable with a median of 2 (IQR = 1 and 2, respectively) in both groups. Most patients used lamotrigine or lacosamide in combination with another AED instead of monotherapy: 66% and 71%, respectively (p = 0.534). Of these combinations, the combination with levetiracetam was most common in both groups (33% and 49% for lamotrigine and lacosamide respectively). Median total daily dosage of lamotrigine versus lacosamide at the moment of treatment failure due to uncontrolled seizures was 200 mg (IQR = 200) versus 250 mg (IQR = 250), p = 0.548. When AED treatment failed due to intolerable AEs, median dosages were 100 mg (IQR = 81) for lamotrigine versus 200 mg (IQR = 100) for lacosamide, p = 0.131.

### Treatment failure rate

**Fig. 1:**
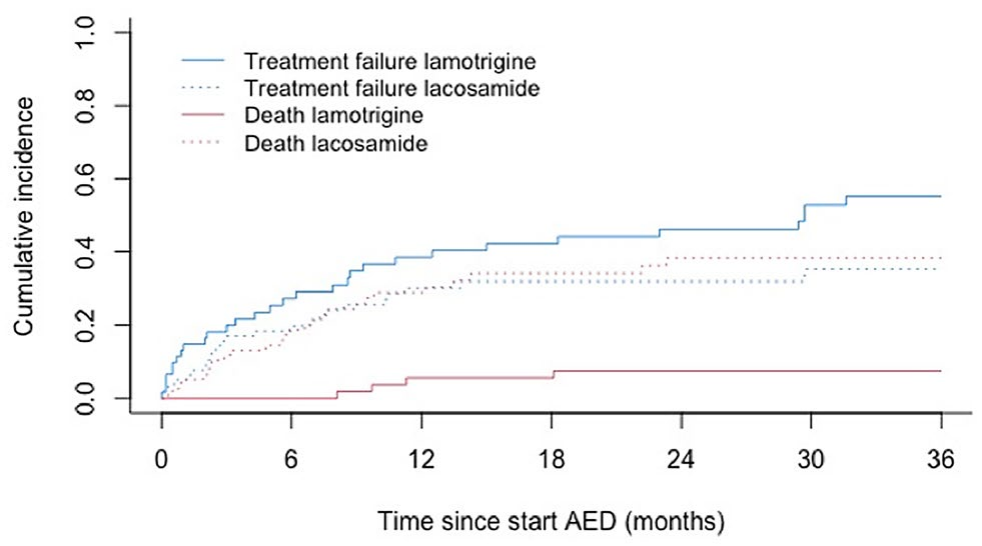
Treatment failure rates for any reason (bold lines) and death: lamotrigine versus lacosamide

The cumulative incidence of treatment failure for any reason showed no significant difference between lamotrigine and lacosamide: 38% (95%CI 26–51%) versus 30% (95%CI 20–41%) (Fig. 1 and Table 2). AED treatment was not independently associated with treatment failure in multivariable analyses: hazard ratio of 0.84 (95%CI 0.46–1.56) (see Table 3). There was also no association between AED treatment and death during follow-up with an adjusted hazard ratio of 1.63 (95%CI 0.51–5.26) (see Table 4).

**Table 2:** Cumulative incidence functions for treatment for any reason and death

Time in months	0	3	6	12	24	36	p-value
No. at risk							
Lamotrigine, no	61	45	39	30	23	0	
Lacosamide, no	78	52	44	27	14	0	
No. censored							
Lamotrigine, no	0	4	6	6	8	27	
Lacosamide, no	0	4	6	8	15	28	
Event treatment failure for any reason							0.072
CIF (95%CI), lamotrigine	2 (0–8)	20 (11–31)	27 (17–39)	38 (26–51)	46 (32–59)	55 (40–68)	
CIF (95%CI), lacosamide	0 (NA)	17 (10–26)	18 (11–28)	30 (20–41)	32 (21–43)	35 (23–47)	
Event death							< 0.001
CIF (95%CI), lamotrigine	0 (NA)	0 (NA)	0 (NA)	6 (1–14)	7 (2–17)	7 (2–17)	
CIF (95%CI), lacosamide	0 (NA)	12 (6–20)	19 (11–28)	29 (19–40)	38 (26–50)	38 (26–50)	

*CI* confidence interval; *CIF* cumulative incidence function; *NA* not available; *No.* number of patients

**Table 3:** Cause specific hazard ratios along with their 95%CI for time to treatment failure for any reason (univariate and multivariable analysis): a competing risk model with 2 events: failure and death

Parameter^a^		Treatment failure for any reason			
		uHR (95%CI)	p-value	aHR (95%CI)	p-value
AED treatment	Lamotrigine (ref.)				
	Lacosamide	0.79 (0.46–1.35)	0.384	0.84 (0.46–1.56)	0.587
Age	≤ 40 year (ref.)				
	> 40 year	1.34 (0.75–2.40)	0.318	1.50 (0.79–2.88)	0.219
Gender	Male (ref.)				
	Female	1.48 (0.87–2.53)	0.151	1.38 (0.78–2.41)	0.266
Tumor grade	Low grade (ref.)				
	High grade	1.53 (0.89–2.63)	0.126	1.45 (0.75–2.78)	0.268
Surgical resection	No (incl. biopsy) (ref.)				
	Yes	0.99 (0.54–1.79)	0.968	1.08 (0.56–2.10)	0.812
Radiotherapy	No (ref.)				
	Yes	0.95 (0.55–1.63)	0.838	0.91 (0.41–2.02)	0.817
Chemotherapy	No (ref.)				
	Yes	0.94 (0.54–1.61)	0.808	0.89 (0.41–1.96)	0.776
Progressive disease	No (ref.)				
	Yes, > 3 months	1.94 (1.06–3.53)	0.031	0.88 (0.42–1.86)	0.745
	Yes, ≤ 3 months	1.03 (0.50–2.12)	0.930	0.79 (0.38–1.65)	0.793

*AED* Antiepileptic drug; *aHR* adjusted hazard ratio; *CI* confidence interval; *uHR* unadjusted hazard ratio

^a^Seizure type, tumor involvement in the frontal lobe and isocitrate dehydrogenase (IDH)-mutation were stratified because total number of events was 54, resulting in a maximum number of ten parameters

**Table 4:** Cause specific hazard ratios along with their 95%CI for death during follow-up (univariate and multivariable analysis): a competing risk model with 2 events: death and failure

Parameter^a^		Death during follow-up			
		uHR (95%CI)	p-value	aHR (95%CI)	p-value
AED treatment^b^	Lamotrigine (ref.)				
	Lacosamide	1.76 (0.60–5.11)	0.301	1.63 (0.51–5.26)	0.410
Age	≤ 40 year (ref.)				
	> 40 year	0.97 (0.29–3.27)	0.956	0.98 (0.21–4.68)	0.982
Tumor grade	Low grade (ref.)				
	High grade	1.22 (0.49–3.03)	0.676	1.15 (0.33–3.99)	0.824
Progressive disease	No (ref.)				
	Yes, > 3 months	1.26 (0.44–3.64)	0.669	1.19 (0.38–3.71)	0.770
	Yes, ≤ 3 months	1.62 (0.56–4.69)	0.373	1.30 (0.37–4.58)	0.687

*AED* Antiepileptic drug; *aHR* adjusted hazard ratio; *CI* confidence interval; *KPS* Karnofsky Performance Score; *uHR* unadjusted hazard ratio; *?* Unknown; ^*a*^ Improvement after discontinuation of the current therapy with lamotrigine or lacosamide; *CTCAE* Common Terminology Criteria for Adverse Events; *No*. Number of patients

^a^Parameters were selected based on clinical significance

^b^AED treatment did not hold Schoenfeld residuals

### Level of efficacy

Having uncontrolled seizures was the main reason for patients to show AED treatment failure: 25% (15/61) of patients in the lamotrigine group versus 13% (10/78) of patients in the lacosamide group during the 36-month follow-up period. The cumulative incidence of treatment failure due to uncontrolled seizures at 12 months was 18% (95%CI 9–29%) for lamotrigine and 11% (95%CI 5–20%) for lacosamide. Supplemental S2 shows the cumulative incidence for specific treatment failure reasons and death; details can be found in Supplemental S3.

### Level of toxicity

A total of 25 patients experienced treatment failure due to one (*n* = 18), two (*n* = 4), three (*n* = 1) or four (*n* = 2) AEs, encompassing 37 AEs reported in total within 36 months of follow-up. In the lamotrigine group, 18 AEs in 11 patients were observed which led to treatment failure, which was 19 AEs in 14 patients on lacosamide. Of all reported AEs, agitation (5/37), depression (4/37) and headache (3/37) were reported most often. Of the patients with agitation, 3/5 were on lamotrigine, as were 1/4 patients with depression and 1/3 patients with headache on lamotrigine. In both groups, most of the AEs occurred within the first three months (Supplemental S2). The cumulative incidences of treatment failure due to AEs at 12 months were 17% (95%CI 9–28%) and 19% (95%CI 11–29) for lamotrigine and lacosamide, respectively. Of all reported AEs, grade 3 or 4 counted for 17% (3/18) in the lamotrigine group, whereas no (0/19) grade 3 or 4 AEs were reported in the lacosamide group (p = 0.264). Improvement of AEs after discontinuation of lamotrigine or lacosamide occurred in 72% of all grade AEs (13/18) in the lamotrigine group, compared to 53% (10/19) in the lacosamide group (p = 0.083) (Supplemental S4 for detailed information on AEs).

## Discussion

So far, no studies have compared the effectiveness of lamotrigine versus lacosamide in glioma patients with epilepsy. We show that lamotrigine and lacosamide are equally effective. The cumulative incidence of treatment failure for any reason was 30% and 38% for lacosamide and lamotrigine, respectively, and multivariable analysis did not show an independent association between AED treatment and treatment failure. Comparable results were found for secondary outcomes of treatment failure due to uncontrolled seizures and level of toxicity, showing no significant differences between the AEDs.

Lamotrigine and lacosamide, a second and third generation AED, respectively, previously showed to be effective and well tolerated in patients with non-BTRE. Lamotrigine had improved efficacy over frequently used second-line AEDs like carbamazepine for time to treatment failure for any reason [15, 16, 28, 29]. Lacosamide demonstrated relatively high effectiveness as add-on therapy in BTRE, with 6-month retention rates up to 86% [19–21, 30, 31]. The anticonvulsive action mechanism of lamotrigine, a voltage-gated sodium channel blocker, is due to the inhibition of glutamate excitotoxicity [32]. It is metabolized primarily by glucuronidation and it has a half-life of approximately 30 hours, although this is shortened to 14 hours in the concomitant use of enzyme-inducing AEDs like carbamazepine and phenytoin, or may be prolonged, depending on the dosages, in the combination with valproic acid, a glucuronidation inhibitor [33]. This interaction should be taken into account when prescribing lamotrigine to a patient who concomitantly uses valproic acid. Lacosamide acts as a slow inactivator of voltage-gated sodium channels and has a half-life of approximately 13 hours with a low potential for drug-drug interactions [32]. Our data set suggests that these two AEDs are regularly used in clinical practice after levetiracetam and/or valproic acid failed as first-line AED treatment, which corresponds to international recommendations [11, 34].

In our study, cumulative incidence rates for death were significantly higher in the lacosamide group compared to the lamotrigine group (i.e. 29 versus 6%). This difference might reflect a bias towards a worse prognosis at baseline for patients on lacosamide compared to those on lamotrigine. This is supported by the fact that more patients on lacosamide had progressive disease both before and after initiation of the AED and more frequently had received radio- and/or chemotherapy, compared to patients on lamotrigine. Overrepresentation of patients with a low-grade glioma in the lamotrigine group might also be a result of the fact that lamotrigine is a much older AED regimen, leading to a relative accumulation of patients with a favorable survival being treated with lamotrigine compared to lacosamide. Additionally, lamotrigine needs a careful titration in several weeks before effective dosages can be reached. In patients with high-grade gliomas in whom time is often limited, physicians may be inclined to prefer lacosamide above lamotrigine to ensure a rapid initiation of AED treatment. Moreover, no association between AED treatment and death was observed in the competing risk model for death during follow-up. Together with the lack of evidence for possible drug-related death in patients who used lacosamide [35–37], it seems implausible lacosamide has an effect on overall survival.

This study has some limitations. First of all, its retrospective nature together with the relatively small sample size hampers the conclusions that can be drawn since recall and report bias cannot be excluded as well as residual confounding. Secondly, due to their poor prognosis and stage of disease at baseline many glioma patients were lost to follow-up or deceased, resulting in only 30 patients at risk in the lamotrigine group and 27 in the lacosamide group at 12 months. Thirdly, two-thirds of the patients in this study were on polytherapy with either lamotrigine or lacosamide, reflecting the heterogeneity of the population. In addition, due to the small sample sizes we considered subgroup analyses to be inappropriate. Future studies will likely have a similar bias given the wide range of AEDs available for patients with epilepsy. Creating sufficiently large sample sizes for adequate comparisons of two AEDs would require enormous data sets. Nevertheless, we believe the ratio between mono- and polytherapy in our study is a realistic reflection of today’s clinical practice. Fourthly, the relatively small sample sizes in this study have also resulted in the inability to calculate cause specific hazard ratios for the secondary outcomes.

In conclusion, this retrospective observational cohort study showed no significant difference in terms of effectiveness between lamotrigine and lacosamide when used after failure on first-line AEDs. Treatment failure rates due to uncontrolled seizures and due to intolerable AEs were similar between the two groups. Therefore, lamotrigine and lacosamide seem to be comparable in terms of efficacy and tolerability in glioma patients with epilepsy who have failed on first-line AEDs. Future prospective randomized controlled trials should focus on providing further evidence for the best AED treatment strategy in patients with BTRE, not only regarding effectiveness but also on health-related quality of life and psychiatric symptoms.

## Supplementary Information

Below is the link to the electronic supplementary material.

Supplementary file1 (DOCX 177 kb)

## Data Availability

The datasets generated during and/or analysed during the current study are available from the corresponding author on reasonable request.

## References

[1] Wang J, Hu G, Quan X (2019). Analysis of the factors affecting the prognosis of glioma patients. Open Med.

[2] Sun J, Shi H, Lai N, Liao K, Zhang S, Lu X (2014). Overexpression of microRNA-155 predicts poor prognosis in glioma patients. Med Oncol.

[3] Fuller GN (2008). The WHO classification of tumours of the central nervous system, 4th edition. Arch Pathol Lab Med.

[4] Nieder C, Adam M, Molls M, Grosu AL (2006). Therapeutic options for recurrent high-grade glioma in adult patients: recent advances. Crit Rev Oncol Hematol.

[5] Phan K, Ng W, Lu VM, McDonald KL, Fairhall J, Reddy R (2018). Association between IDH1 and IDH2 mutations and preoperative seizures in patients with low-grade versus high-grade glioma: a systematic review and meta-analysis. World Neurosurg.

[6] Englot DJ, Chang EF, Vecht CJ (2016). Epilepsy and brain tumors. Handb Clin Neurol.

[7] Chen DY, Chen CC, Crawford JR, Wang SG (2018). Tumor-related epilepsy: epidemiology, pathogenesis and management. J Neurooncol.

[8] Vecht C, Royer-Perron L, Houillier C, Huberfeld G (2017). Seizures and anticonvulsants in brain tumours: frequency, mechanisms and anti-epileptic management. Curr Pharm Des.

[9] Maschio M, Aguglia U, Avanzini G, Banfi P, Buttinelli C, Capovilla G (2019). Management of epilepsy in brain tumors. Neurol Sci.

[10] You G, Sha ZY, Yan W, Zhang W, Wang YZ, Li SW (2012). Seizure characteristics and outcomes in 508 Chinese adult patients undergoing primary resection of low-grade gliomas: a clinicopathological study. Neuro Oncol.

[11] Vecht CJ, Kerkhof M, Duran-Pena A (2014). Seizure prognosis in brain tumors: new insights and evidence-based management. Oncologist.

[12] Maschio M, Beghi E, Casazza MML, Colicchio G, Costa C, Banfi P (2017). Patterns of care of brain tumor-related epilepsy. A cohort study done in Italian epilepsy center. PLoS ONE.

[13] van der Meer PB, Dirven L, Fiocco M, Vos MJ, Kouwenhoven MCM, van den Bent MJ (2021). First-line antiepileptic drug treatment in glioma patients with epilepsy: levetiracetam vs valproic acid. Epilepsia..

[14] Panebianco M, Bresnahan R, Ramaratnam S, Marson AG (2020). Lamotrigine add-on therapy for drug-resistant focal epilepsy. Cochrane Database Syst Rev.

[15] Fakhoury TA, Hammer AE, Vuong A, Messenheimer JA (2004). Efficacy and tolerability of conversion to monotherapy with lamotrigine compared with valproate and carbamazepine in patients with epilepsy. Epilepsy Behav.

[16] Kaminow L, Schimschock JR, Hammer AE, Vuong A (2003). Lamotrigine monotherapy compared with carbamazepine, phenytoin, or valproate monotherapy in patients with epilepsy. Epilepsy Behav.

[17] Nevitt SJ, Sudell M, Weston J, Tudur Smith C, Marson AG (2017). Antiepileptic drug monotherapy for epilepsy: a network meta-analysis of individual participant data. Cochrane Database Syst Rev.

[18] Glauser T, Ben-Menachem E, Bourgeois B, Cnaan A, Guerreiro C, Kälviäinen R (2013). Updated ILAE evidence review of antiepileptic drug efficacy and effectiveness as initial monotherapy for epileptic seizures and syndromes. Epilepsia.

[19] Rudà R, Pellerino A, Franchino F, Bertolotti C, Bruno F, Mo F (2018). Lacosamide in patients with gliomas and uncontrolled seizures: results from an observational study. J Neurooncol.

[20] Maschio M, Zarabla A, Maialetti A, Fabi A, Vidiri A, Villani V (2017). Quality of life, mood and seizure control in patients with brain tumor related epilepsy treated with lacosamide as add-on therapy: a prospective explorative study with a historical control group. Epilepsy Behav.

[21] Rudà R, Houillier C, Maschio M, Reijneveld JC, Hellot S, De Backer M (2020). Effectiveness and tolerability of lacosamide as add-on therapy in patients with brain tumor-related epilepsy: Results from a prospective, noninterventional study in European clinical practice (VIBES). Epilepsia.

[22] Glauser T, Ben-Menachem E, Bourgeois B, Cnaan A, Chadwick D, Guerreiro C (2006). ILAE treatment guidelines: evidence-based analysis of antiepileptic drug efficacy and effectiveness as initial monotherapy for epileptic seizures and syndromes. Epilepsia.

[23] van der Meer PB, Dirven L, Fiocco M, Taphoorn MJ, Koekkoek JA (2020). Retention rates of antiepileptic drugs in glioma patients: the most appropriate outcome. CNS Oncol.

[24] UdoHaH. S Common Terminology Criteria for Adverse Events (CTCAE) version 5.0 [online] 2017 [Available from: https://ctep.cancer.gov/protocoldevelopment/electronic_applications/docs-/CTCAE_v5_Quick_Reference_8.5x11.pdf. Accessed 21 Aug 2020

[25] WHO Collaborating Centre for Drug Statistics Methodology. ATC/DDD Index 2021., from: http://www.whocc.no/atcddd/. Accessed 19 Apr 2021

[26] Putter H, Fiocco M, Geskus RB (2007). Tutorial in biostatistics: competing risks and multi-state models. Stat Med.

[27] Gray RJ (1988). A class of K-sample tests for comparing the cumulative incidence of a competing risk. Ann Stat.

[28] Nevitt SJ, Tudur Smith C, Weston J, Marson AG (2018). Lamotrigine versus carbamazepine monotherapy for epilepsy: an individual participant data review. Cochrane Database Syst Rev.

[29] Marson AG, Al-Kharusi AM, Alwaidh M, Appleton R, Baker GA, Chadwick DW (2007). The SANAD study of effectiveness of carbamazepine, gabapentin, lamotrigine, oxcarbazepine, or topiramate for treatment of partial epilepsy: an unblinded randomised controlled trial. Lancet.

[30] Saria MG, Corle C, Hu J, Rudnick JD, Phuphanich S, Mrugala MM (2013). Retrospective analysis of the tolerability and activity of lacosamide in patients with brain tumors: clinical article. J Neurosurg.

[31] Maschio M, Dinapoli L, Mingoia M, Sperati F, Pace A, Pompili A (2011). Lacosamide as add-on in brain tumor-related epilepsy: preliminary report on efficacy and tolerability. J Neurol.

[32] Sills GJ, Rogawski MA (2020). Mechanisms of action of currently used antiseizure drugs. Neuropharmacology..

[33] Kanner AM, Frey M (2000). Adding valproate to lamotrigine: a study of their pharmacokinetic interaction. Neurology.

[34] Rudà R, Soffietti R (2015). What is new in the management of epilepsy in gliomas?. Curr Treat Options Neurol.

[35] Vossler DG, Knake S, O'Brien TJ, Watanabe M, Brock M, Steiniger-Brach B (2020). Efficacy and safety of adjunctive lacosamide in the treatment of primary generalised tonic-clonic seizures: a double-blind, randomised, placebo-controlled trial. J Neurol Neurosurg Psychiatry.

[36] Steinhoff BJ, Eckhardt K, Doty P, De Backer M, Brunnert M, Schulze-Bonhage A (2016). A long-term noninterventional safety study of adjunctive lacosamide therapy in patients with epilepsy and uncontrolled partial-onset seizures. Epilepsy Behav.

[37] Paquette V, Culley C, Greanya ED, Ensom MH (2015). Lacosamide as adjunctive therapy in refractory epilepsy in adults: a systematic review. Seizure.

