# Supplementary material for: The effectiveness of antiepileptic drug treatment in glioma patients: lamotrigine versus lacosamide
Source: J Neurooncol. 2021 Jul 1;154(1):73–81. doi: 10.1007/s11060-021-03800-z (PMC8367894; doi:10.1007/s11060-021-03800-z)
Supplement: Supplementary file 1 — Supplementary file1 (DOCX 177 kb) [file 11060_2021_3800_MOESM1_ESM.docx]

*Supplemental S1: Non-response analysis*

|  | **Lamo/laco**  ***n = 139*** | **Other**  ***n = 1296*** | **p-value** |
| --- | --- | --- | --- |
| Gender, male, no. (%) | 81 (58%) | 851 (66%) | 0.083 |
| Mean age (SD) | 48.0 (13.3) | 53.7 (14.5) | <0.001 |
| Age group, no. (%) |  |  | 0.035 |
| ≤ 40 year | 39 (28%) | 264 (20%) |  |
| > 40 year | 100 (72%) | 1032 (80%) |  |
| KPS, no. (%) |  |  | <0.001 |
| ≥70 | 132 (95%) | 1203 (93%) |  |
| <70 | 5 (4%) | 93 (7%) |  |
| Unknown | 2 (1%) | 0 (0%) |  |
| WHO diagnosis (%) |  |  | <0.001 |
| Grade 2 |  |  |  |
| Diffuse astrocytoma NOS | 17 (12%) | 100 (8%) |  |
| Diffuse astrocytoma IDH-mutant | 17 (12%) | 66 (5%) |  |
| Oligodendroglioma NOS | 12 (9%) | 45 (4%) |  |
| Oligodendroglioma IDH-mutant  1p/19q codeletion | 14 (10%) | 81 (6%) |  |
| Oligoastrocytoma NOS | 2 (1%) | 13 (1%) |  |
| Pleiomorphic xanthoastrocytoma | 0 (0%) | 3 (0.2%) |  |
| Grade 3 |  |  |  |
| Anaplastic astrocytoma NOS | 7 (5%) | 60 (5%) |  |
| Anaplastic astrocytoma  IDH-mutant | 5 (4%) | 19 (2%) |  |
| Anaplastic oligodendroglioma NOS | 7 (5%) | 35 (3%) |  |
| Anaplastic oligodendroglioma  IDH-mutant 1p/19q codeletion | 4 (3%) | 25 (2%) |  |
| Anaplastic oligoastrocytoma NOS | 1 (1%) | 4 (0%) |  |
| Grade 4 |  |  |  |
| Diffuse astrocytoma wildtype | 4 (3%) | 24 (2%) |  |
| Anaplastic astrocytoma wildtype | 4 (3%) | 14 (1%) |  |
| Glioblastoma NOS | 22 (16%) | 600 (46%) |  |
| Glioblastoma wildtype | 21 (15%) | 187 (14%) |  |
| Glioblastoma IDH-mutant | 2 (1%) | 20 (2%) |  |

No.=number of patients; SD=standard deviation; KPS=Karnofsky Performance Status; WHO=World Health Organization.

No.=number of patients; SD=standard deviation; KPS=Karnofsky Performance Status; WHO=World Health Organization.


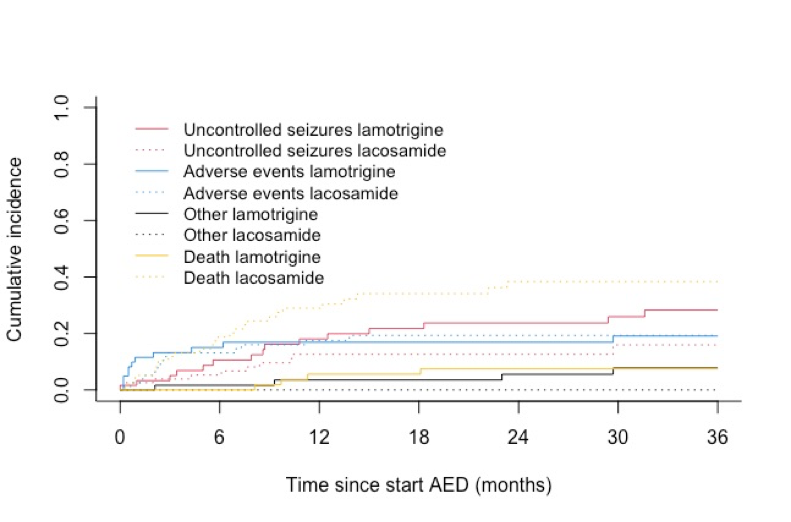


*Supplemental S2: Estimated cumulative incidences corresponding to each competing event for the groups lamotrigine and lacosamide*

*Supplemental S3: Cumulative incidence functions for treatment failures and death*

| **Time in months** | 0 | 3 | 6 | 12 | 24 | 36 | p-value |
| --- | --- | --- | --- | --- | --- | --- | --- |
| **No. at risk** | | | | | | |  |
| Lamotrigine, no. | 61 | 45 | 39 | 30 | 23 | 0 |  |
| Lacosamide, no. | 78 | 52 | 44 | 27 | 14 | 0 |  |
| **No. censored** | | | | | | |  |
| Lamotrigine, no. | 0 | 4 | 6 | 6 | 8 | 27 |  |
| Lacosamide, no. | 0 | 4 | 6 | 8 | 15 | 28 |  |
| **Treatment failure** | | | | | | |  |
| *Event uncontrolled seizures* | | | | | | | 0.088 |
| CIF (95%CI), lamotrigine | 2 (0-8) | 5 (1-13) | 11 (4-20) | 18 (9-29) | 24 (13-36) | 28 (17-41) |  |
| CIF (95%CI), lacosamide | 0 (NA) | 4 (1-10) | 5 (2-12) | 11 (5-20) | 11 (5-20) | 14 (6-25) |  |
| *Event adverse events* | | | | | | | 0.876 |
| CIF (95%CI), lamotrigine | 0 (NA) | 13 (6-23) | 15 (7-25) | 17 (9-28) | 17 (9-28) | 19 (10-30) |  |
| CIF (95%CI), lacosamide | 0 (NA) | 13 (7-22) | 13 (7-22) | 19 (11-29) | 21 (12-31) | 21 (12-31) |  |
| *Event other reasons^1^* | | | | | | | 0.041 |
| CIF (95%CI), lamotrigine | 0 (NA) | 2 (0-8) | 2 (0-8) | 4 (1-11) | 6 (1-14) | 8 (2-17) |  |
| CIF (95%CI), lacosamide | 0 (NA) | 0 (NA) | 0 (NA) | 0 (NA) | 0 (NA) | 0 (NA) |  |
| **Event death** | | | | | | | <0.001 |
| CIF (95%CI), lamotrigine | 0 (NA) | 0 (NA) | 0 (NA) | 6 (1-14) | 7 (2-17) | 7 (2-17) |  |
| CIF (95%CI), lacosamide | 0 (NA) | 12 (6-20) | 19 (11-28) | 29 (19-40) | 38 (26-50) | 38 (26-50) |  |

^1^Other encompassed withdrawal due to remission (i.e. discontinuation of the antiepileptic drugs with consent of the physician, *n*=3) and unknown reasons (*n*=1); CI=confidence interval; CIF=cumulative incidence function; NA=not available; No.=number of patients

*Supplemental S4: Detailed overview of adverse effects which led to treatment failure*

| **Adverse Effects According to the CTCAE 5.0** | **Lamotrigine** | | | | | | | **Lacosamide** | | | | | | |
| --- | --- | --- | --- | --- | --- | --- | --- | --- | --- | --- | --- | --- | --- | --- |
|  | **Grade, no.** | | | | **Improved, no.**^1^ | | | **Grade, no.** | | | | **Improved, no.** | | |
|  | **I & II** | **III & IV** | **?** | **Total** | **Yes** | **No** | **?** | **I & II** | **III & IV** | **?** | **Total** | **Yes** | **No** | **?** |
| **Blood and lymphatic system disorders** | | | | | | | |  | | | | | | |
| Lymphadenopathy | - | - | 1 | 1 | 1 | - | - | - | - | - | 0 | - | - | - |
| **Gastrointestinal disorders** | | | | | | | |  | | | | | | |
| Dyspepsia | - | - | - | 0 | - | - | - | 1 | - | - | 1 | 1 | - | - |
| **General and administration site conditions** | | | | | | | |  | | | | | | |
| Clinical deterioration | - | - | - | 0 | - | - | - | 1 | - | - | 1 | - | - | 1 |
| Fatigue | 1 | - | - | 1 | 1 | - | - | - | - | - | 0 | - | - | - |
| **Metabolism and nutrition disorders** | | | | | | | |  | | | | | | |
| Anorexia | - | - | - | 0 | - | - | - | 1 | - | - | 1 | - | 1 | - |
| **Nervous system disorders** | | | | | | | |  | | | | | | |
| Ataxia | 1 | - | - | 1 | 1 | - | - | - | - | - | 0 | - | - | - |
| Concentration impairment | 1 | - | - | 1 | 1 | - | - | 2 | - | - | 2 | 1 | 1 | - |
| Dizziness | 2 | - | - | 2 | 1 | - | 1 | - | - | - | 0 | - | - | - |
| Encephalopathy | - | 1 | - | 1 | 1 | - | - | - | - | - | 0 | - | - | - |
| Headache | 1 | - | - | 1 | 1 | - | - | 2 | - | - | 2 | - | 2 | - |
| Memory impairment | - | - | - | 0 | - | - | - | 1 | - | - | 1 | - | 1 | - |
| Paresthesia | 1 | - | - | 1 | 1 | - | - | - | - | - | 0 | - | - | - |
| Presyncope | - | - | - | 0 | - | - | - | 1 | - | - | 1 | 1 | - | - |
| Somnolence | 1 | - | - | 1 | - | - | 1 | 1 | - | - | 1 | 1 | - |  |
| Tremor | 1 | - | - | 1 | 1 | - | - | - | - | - | 0 | - | - | - |
| **Psychiatric disorders** | | | | | | | |  | | | | | | |
| Agitation | 3 | - | - | 3 | 2 | - | 1 | 2 | - | - | 2 | 1 | 1 | - |
| Depression | - | 1 | - | 1 | - | 1 | - | 3 | - | - | 3 | 2 | 1 | - |
| Hallucinations | 1 | - | - | 1 | 1 | - | - | - | - | - | 0 | - | - | - |
| Insomnia | - | - | - | 0 | - | - | - | 1 | - | - | 1 | 1 | - | - |
| Psychosis | - | 1 | - | 1 | - | 1 | - | - | - | - | 0 | - | - | - |
| **Skin and subcutaneous tissue disorders** | | | | | | | |  | | | | | | |
| Rash | 1 | - | - | 1 | 1 | - | - | 1 | - | - | 1 | - | 1 | - |
| **Unknown** | | | | | | | |  | | | | | | |
| Unknown | - | - | - | 0 | - | - | - | - | - | 2 | 2 | 2 | - | - |
| **Total all adverse effects** | 14 | 3 | 1 | 18 | 13 | 2 | 3 | 17 | 0 | 2 | 19 | 10 | 8 | 1 |
